# Informing a Randomized Control Trial in Rural Populations: Adaptation of a Diabetes Self-Management Education and Support Intervention

**DOI:** 10.2196/35664

**Published:** 2022-06-10

**Authors:** Tamara K Oser, Linda Zittleman, Kristen Curcija, Bethany Kwan, Shawnecca Burke, Sindy Gonzalez, Kelsey Huss, Marilee Johnson, Norah Sanchez, Julie Neuberger, Eli Iacob, Juliana Simonetti, Michelle Litchman

**Affiliations:** 1 Department of Family Medicine School of Medicine University of Colorado Aurora, CO United States; 2 Community Advisory Council High Plains Research Network Aurora, CO United States; 3 Department of Internal Medicine University of Utah Salt Lake City, UT United States; 4 College of Nursing University of Utah Salt Lake City, UT United States

**Keywords:** diabetes, self-management, diabetes self-management education and support (DSMES), rural health, boot camp translation (BCT), community medicine

## Abstract

**Background:**

Over 34 million people in the United States have diabetes, with 1.5 million diagnosed every year. Diabetes self-management education and support (DSMES) is a crucial component of treatment to delay or prevent complications. Rural communities face many unique challenges in accessing DSMES, including geographic barriers and availability of DSMES programs that are culturally adapted to rural context.

**Objective:**

Boot Camp Translation (BCT) is an established approach to community-based participatory research used to translate complex clinical and scientific information into concepts, messages, and materials that are understandable, meaningful, and relevant to community members and patients. This study aimed to utilize BCT to adapt an existing DSMES program for delivery in rural primary care for English- and Spanish-speaking people with diabetes.

**Methods:**

The High Plains Research Network (HPRN) Community Advisory Council (C.A.C.) partnered with researchers at the University of Colorado and University of Utah to use BCT to aid in translating medical jargon and materials from an existing DSMES program, called “Diabetes One Day (D1D).” BCT consisted of 10 virtual meetings over a 6-month period among the C.A.C., which included 15 diverse community stakeholders. Both English-speaking and bilingual Spanish-English–speaking C.A.C. members were recruited to reflect the diversity of the rural communities in which the adapted program would be delivered.

**Results:**

The BCT process guided adaptations to D1D for use in rural settings (R-D1D). R-D1D adaptations reflect both content and delivery to assure that the intervention is appropriate and likely to be accepted by rural English- and Spanish-speaking people with diabetes. Additionally, BCT informed the design of recruitment and program materials and identification of recruitment venues. During the BCT process, the importance of tailoring materials to reflect culture differences in English- and Spanish-speaking patients was identified.

**Conclusions:**

BCT was an effective strategy for academic researchers to partner with rural community members to adapt an existing DSMES intervention for delivery in rural areas to both English- and Spanish-speaking patients with diabetes. Through BCT, adaptations to recruitment materials and methods, program content and delivery, and supplemental materials were developed. The need to culturally adapt Spanish materials with input from stakeholders rather than simply translate materials into Spanish was highlighted. The importance of increasing awareness of the connection between diabetes and depression or diabetes distress, adaptations to include local foods, and the importance of the relationship between people with diabetes and their primary care practices were identified.

## Introduction

Diabetes is a chronic, progressive disease affecting over 34 million people and is the 7th leading cause of the death in the United States [1]. Diabetes contributes to serious micro- and macrovascular complications leading to disability and poor quality of life [2]. Diabetes self-management is the cornerstone of avoiding or delaying diabetes complications. Self-management behaviors include a challenging daily diet, medication, exercise, and a glucose monitoring regimen. Diabetes self-management education and support (DSMES) is needed to help people with diabetes adopt self-management behaviors and is recommended by the American Association of Diabetes Educators and the American Diabetes Association as standard of care [3]. DSMES can improve outcomes in both type 1 diabetes (T1D) and type 2 diabetes (T2D), including lower glycated hemoglobin (HbA_1C_), improved quality of life, and healthy coping [4-7]. However, disparities in access to high-quality DSMES are influenced by social determinants of health, leading to downstream health inequity in diabetes outcomes. Notably, there is a lack of culturally appropriate, local DSMES for those who live in rural areas and those who do not speak English, which contributes to higher rates of diabetes-related complications in this population relative to those who live in more urban areas [8,9].

To address the need for improved DSMES, a team led by a nurse-practitioner researcher developed the Diabetes One Day (D1D) Program, an interdisciplinary DSMES program for patients with diabetes and their care partners that enhances diabetes distress and diabetes self-care behaviors through several strategies. The in-person 1-day (8-hour) DMSES intervention provided a hybrid education program that included individual and small group sessions delivered by interdisciplinary diabetes specialists (eg, certified diabetes care and education specialist, pharmacist, licensed clinical social worker, chef). Topics included pathophysiology, medications, weight management, exercise and being active, healthy eating, troubleshooting glucose levels, diabetes technology, and coping with diabetes. Each particiant was encouraged to bring a care partner (family or friend). Sessions were interactive, allowing for peer support. Participants also received written and digital education materials. D1D has been shown to be feasible to deliver through a remote team and to significantly reduce HbA_1C_ in participants seen at an academic endocrinology center [10].

In rural eastern Colorado, rates of diabetes average 12.3% compared with the state average of 7.3% [11]. Clinicians and practice staff in the High Plains Research Network (HPRN) and the HPRN Community Advisory Council (C.A.C.) consistently identify diabetes and the lack of diabetes management support as health priorities during visits to practices and annual research convocations. The HPRN C.A.C. identified the D1D Program as a potential resource to help address disparities in diabetes prevalence and outcomes in rural eastern Colorado. However, existing DSMES programs might rely on resources that are not available in rural regions. Factors that influence program fit with rural primary care and communities include access to practice staff with the training, resources, and time to provide DSMES; patient education materials that do not reflect the social and physical environment (eg, access to sidewalks, fitness centers, a diversity of restaurants and large grocery stores); and cultural and technological infrastructure needs.Therefore, the HPRN C.A.C. and academic research team sought to review and adapt the D1D program for rural eastern Colorado. Boot Camp Translation (BCT) is an evidence-based participatory community engagement method [12] that diverse populations have used to translate scientific evidence-based guidelines into new, locally relevant messages, materials, and dissemination strategies that use local assets [13-18]. For this study, the community-academic partnership used BCT to adapt the existing D1D program delivery method and materials to help increase access to effective DSMES for English- and Spanish-speaking patients, caregivers, and primary care practices in rural eastern Colorado.

We describe the use of BCT to adapt the D1D and the resulting messages and materials that created the Rural Diabetes One Day (R-D1D) program. Results are useful to patients, clinical teams, and researchers in other rural regions lacking DSMES across the United States, as the adaptations may apply to other rural communities outside of rural Colorado, particularly in the Western region of the United States.

## Methods

### Context and Setting

The study was conducted in northeast Colorado, which is part of the HPRN. The HPRN is a primary care- and community-based research network in 16 counties in rural eastern Colorado. The network is housed in the University of Colorado Department of Family Medicine. Of the 16 counties in the HPRN region, 15 counties are in a geographic or income-based primary care health professional shortage area, and approximately 27% of the population is Hispanic, with 12% of the population speaking Spanish at home [19]. Only 5 diabetes care and education specialists are located in the entire 16-county region of the HPRN [20], which covers 30,000 square miles.

### The Diabetes One Day DSMES Program

The D1D Program is an interdisciplinary DSMES program for patients with diabetes and their care partners. D1D was originally designed to be delivered in person over 8 hours in 1 day. Program components are outlined in Textbox 1.

**Adaptations to the Diabetes One Day (D1D) Program structure and content for patients seen in rural primary care practices.**

**Original Diabetes One Day (8 hours)**

1. Diabetes Overview (60 min)
2. Medication Options (30 min)
3. Weight Management and Diabetes (20 min)
4. Importance of Exercise (20 min)
5. Healthy Eating/Carbohydrate Counting (60 min)
6. Meal Demonstration and Lunch (60 min)
7. Individual Visit With Nurse Practitioner/Medical Doctor (90 min)
8. Troubleshooting Glucose Levels (25 min)
9. Diabetes Technology (20 min)
10. Healthy Coping With Diabetes (45 min)

**Rural Diabetes One Day Schedule (5.5 hours)**

1. Coping With Diabetes (60 min)
2. What is Diabetes? (15 min)
3. Diabetes Complications (15 min)
4. Self-Care Behaviors (20 min)
5. Sick Day Management (10 min)
6. Troubleshooting Glucose Levels (20 min)
7. Weight and Diabetes (15 min)
8. Healthy Eating (50 min)
9. Medication Options (20 min)
10. Shared Medical Visit (60 min)
11. How to Work With the Health Care Practitioner Team (10 min)

### Adaptation Using Boot Camp Translation

BCT has been used by partnerships of community members, academic researchers, and health professionals around the country on a range of health topics to translate medical information and clinical guidelines into concepts, messages, and materials that are understandable, meaningful, and engaging to community members and patients and disseminated in testable health interventions [21]. A full description of the standard BCT process has been previously reported [22]. We used the BCT process in this study to modify and adapt the existing D1D program for implementation in rural primary care practices and communities.

Community partners consisted of 15 people from diverse backgrounds. Partners included members of the HPRN C.A.C., including ranchers, a teacher, a retired social worker, an agricultural business manager, a school support staff worker, a practice administrator, and locally based HPRN community research liaison/practice facilitators. Ad hoc members were added to round out expertise and perspectives, including 4 people living with T1D or T2D. The HPRN Director, who practices at one of the participating primary care practices, and co-Director facilitated and participated in all BCT meetings. Of the members, 5 care for someone with diabetes, and 5 were Hispanic or Latino, the latter which were all bilingual English-Spanish speaking. Participants also included 3 research partners from the University of Utah who developed the D1D program. A clinician with expertise in diabetes who identifies as Latino and is Spanish-speaking provided the educational presentation and clinical guidance throughout the process.

The BCT process occurred over a 6-month period. Due to the COVID-19 pandemic, the BCT was conducted virtually (using Zoom), and the traditional cycle of meetings and calls was slightly modified to accommodate this format. The process started with a 4-hour kick-off meeting with an expert presentation that provided information about diabetes, evidence-based guidelines for DSMES, and description of the current D1D program. Four 2-hour meetings interspersed by five 45-minute meetings were used to determine content (program messages and materials) and context (program delivery mode, structure) adaptations and to develop recruitment materials.

### Ethics Approval

Required institutional review board approvals and data use agreements among participating organizations have been established. Study procedures were approved by The University of Utah Multiple Institutional Review Board on October 28, 2020 (approval #00133179).

## Results

The BCT process resulted in new study recruitment materials and strategies; new and adapted messages and materials for English- and Spanish-speaking patients; and adaptations to the delivery, structure, and content of the DSMES intervention.

### Recruitment Messages and Materials

The D1D Program was originally offered to patients seen at an academic endocrinology center. This study aimed to implement the program in rural primary care settings. Because many rural communities have only 1 primary care practice, the C.A.C. developed materials for distribution in the practice as well as the broader community. Materials included versatile “inserts”—large, bookmark-sized flyers placed on countertops (such as at clinic check-in areas), in exam rooms, in church bulletins, attached to pharmacy and other retail bags, among other locations. “Inserts” were modified to create 11x17 posters to be hung at various locations in practices and communities. Building on social distancing cues related to COVID-19, floor decals were created for exam rooms and other areas at practices. Recruitment materials were designed for both English- and Spanish-speaking patients. The C.A.C. recommended placing recruitment materials at multiple and diverse locations, including the grocery stores, dollar store, butcher, pharmacy, Mexican bakery, school, church, community center, and meat factory where many Spanish-speaking patients with diabetes are employed.

Recruitment materials carried attention-grabbing messages. The need to care for yourself for the sake of your family was identified as a key message for Spanish-speaking communities, resulting in the message “Porque mi familia importa!” The commonly used term “azúcar” refers to diabetes in the Spanish recruitment materials. Community partners stated that the local rural culture often includes a “take care of yourself” approach to health, and that for some conditions, such as diabetes, people believe they did something wrong and feel guilty. In response, materials for English-speaking participants promoted the message that “Eastern Colorado is worth it!” and that “People with diabetes deserve to live a healthy life.” Images of the landscape and people from the local area were used to give materials an authentic rural look and potential face recognition. Multimedia Appendix 1 offers a description, distribution efforts, and image of the materials developed.

### Participant Program Materials

The D1D Program offers multiple resources for participants. These include the Calorie King book, a Healthy Plate handout developed by the Centers for Disease Control and Prevention (CDC), a handout on SMART goals, copies of the presentations (eg, PowerPoint slides) given during the program, and copies of diabetes management magazines. The C.A.C. identified several adaptations to existing materials and new resources with messages they believed would improve the cultural relevance and, in turn, usefulness of the program in the study region. The adaptations are described in the following sections.

### Recipe Book

Many of the foods and restaurants included in the Calorie King book are not available in eastern Colorado, and the book was not available in Spanish. The C.A.C. adapted this concept into a book with local recipes. “From Our Home to Yours” contains recipes collected from the C.A.C. and friends and a series of tips based on evidence-based guidelines and their lived experiences for participants to use while grocery shopping, preparing meals, and eating. Recipes use affordable foods easily available in eastern Colorado and that reflect the local rural and, within that, Hispanic/Latino culture. All recipes were reviewed by a certified dietician, and slight modifications were suggested to reduce carbohydrate or fat content and portion size, as needed. The book also includes a section introducing the concept of carbohydrates. The C.A.C. wrote the forward to include motivational messages reflected in recruitment materials. The book is available in English and Spanish.

### Healthy Plate Place Mats

This rural region is largely ranch land, and beef is a common food and source of income for a substantial number of people in the region. Instead of promoting foods less common in the region, the C.A.C. worked with their academic partners to offer strategies for healthier consumption of beef than people often practice. The image of salmon found in the original Healthy Plate was replaced with lean beef. Healthy lifestyle recommendations and personal stories from the Recipe Book were added surrounding the Healthy Plate. The “handout” format was converted to a place mat with the intention of increasing exposure to the information. The new Healthy Plate place mat includes the plate and tips on one side and eastern Colorado images on the other.

### Diabetes Distress/Mood Matters Handout and Mood Tracker

The D1D program includes a session on mental health, emphasizing realistic diabetes management goals and coping strategies. The C.A.C. determined that R-D1D needed to enhance the content related to mental health and the connection to diabetes. The resulting colorful handout specifically calls out the connection between diabetes and depression and the reality of diabetes distress. The C.A.C. chose to use the word “mood” to make the concept of depression more accessible and potentially less stigmatizing to participants. With the heading “Mood Matters,” the handout includes actionable strategies for talking with clinical care teams about mood changes. For example, for some people, discussing a change in mood in relation to a physical condition might be easier than when framed solely as a mental health issue. The material includes a combination of evidence-based distress coping strategies from the literature reviewed by the C.A.C. and suggestions based on their own experience, which were reviewed by the partnering clinical diabetes experts. The R-D1D materials include a journal with prompting questions to encourage participants (and care partners) to track their mood and talk with their health care team about changes in mood and how that might impact their diabetes.

### Presentation Handouts

The C.A.C. confirmed the importance of each participant having a copy of the presentation slides. Color copies were printed and included in participant packets.

### Cinch Bag

Materials were provided to participants in a tote bag with the simple message “I’m worth it” on one side and “Yo valgo la pena” on the other side. The bag is intended to increase exposure to this simple message among participants and potentially generate conversations with others in the community about diabetes and the R-D1D program.

### Program Delivery, Structure, and Content

D1D was designed to be delivered in person over 8 hours in 1 day. Broad dissemination by the D1D team to other regions of the country requires a virtual platform (eg, Zoom). The C.A.C. deemed the delivery of a telehealth intervention to participant homes acceptable as long as the virtual platform was easy to use. Further, the program was offered such that participants could participate from their home or join other participants at the primary care clinic where they obtain care, using the practice’s video-conferencing technologies. The resulting program followed the practice’s COVID-19 protocols and allowed participants an option based on their preferences or needs.

The C.A.C. was concerned that attrition would be high if R-D1D was delivered as an 8-hour virtual course, and they expressed concern that a 4-hour course would provide an incomplete, unsatisfying educational experience without enough time spent on topics identified as being especially important, such as self-care and coping. The program was thus restructured to last 5.5 hours and include breaks and maintain multiple interactive and experience-sharing components.

Textbox 1 describes adaptations to the original D1D program content. Specifically, the C.A.C. recommended increasing the amount of time devoted to the “Coping with Diabetes” section and emphasizing the link between diabetes and depression, the reality of diabetes distress, and the new Mood Matters handout and Mood Tracker. Rural populations receive mental health treatment for depression and other mood disorders less frequently than urban counterparts [23], and the C.A.C. felt that many in the community were not aware of the link between diabetes and depression or diabetes distress. Thus the C.A.C. recommended starting R-D1D with a session on “Coping with Diabetes,” in addition to lengthening the time spent on this topic and including an additional session on Self-Care Behaviors. Food choices and eating behaviors emerged as a particularly relevant component of successful diabetes self-management. As a result, the Healthy Eating session was retained and bolstered with the addition of the new Recipe Book and Healthy Plate place mat. Although understanding the concept of carbohydrates was determined important, the group removed the section on carbohydrate counting, noting that a general understanding of low and high carbohydrate foods was more relevant in their experiences and higher priorities for the program. The HPRN C.A.C. valued the evidence supporting the effectiveness of medications to help manage diabetes. A 1-hour shared medical visit with a clinician (nurse practitioner or physician) replaced individual meetings with 3 providers. They chose to retain the D1D’s group format with open discussion, based on a positive experience in Utah. Although participants might feel uncomfortable sharing their health information, the C.A.C. recommended this format to tap into their rural community culture of “neighbor helping neighbor” and made clear that R-D1D clinicians allow participants to opt out of group discussion if they prefer. The Importance of Exercise session was not selected to remain as a stand-alone DSMES strategy in the R-D1D. Instead, the new recipe book and Healthy Plate place mats included encouraging messages and tips related to physical activity. The C.A.C. also advised the R-D1D training team to eliminate references to gym memberships due to the severe limited number of gyms in the region. Given the delivery of R-D1D during the COVID-19 pandemic, the C.A.C. recommended the addition of a session on Sick Day Management. To highlight the partnership between R-D1D and local primary care practices, it was recommended to include a session on How to Work With Your Healthcare Team and that referral notes summarizing patient goals and medication recommendations be sent back to the primary care clinician after their patient attended R-D1D.

## Discussion

### Principal Findings

Our community-academic partnership successfully used the BCT process to adapt the original D1D DSMES program to optimize fit in rural primary care and communities, while retaining the essential elements of the intervention.

Built on the principles of community-based participatory research, BCT values and requires an equitable exchange of perspectives and expertise of its community members, academic research, and clinician participants [24]. This creates an environment of co-learning. The HPRN C.A.C. and staff learned more about diabetes, DSMES evidence, and the reasoning behind certain aspects of the original D1D program. The D1D research team learned about factors in this rural region that might influence a patient’s willingness and capacity to receive health information and follow health guidelines (cultural norms, local resources) and ability to receive information (access to technology, literacy, local language). With this information, the D1D research team could understand the rationale behind the C.A.C.’s recommendations and decisions.

This environment of co-learning allowed the group to negotiate several adaptations. For example, the C.A.C. wondered if participants would retain more information over multiple shorter sessions versus 1 extended session. The group reviewed the implementation and clinical evidence (eg, attendance, change in HbA_1c_) that supported the 1-day D1D structure and discussed how attendance often drops substantially during multisession programs. A 1-day structure reduces barriers to attendance, such as the need to adjust one’s work schedule multiple times, find childcare, and, if attending virtually at the local practice, to find transportation or drive a long distance, which is common in rural regions, multiple times. As a result, the group agreed to testing the feasibility of a 5.5-hour, 1-day program. The importance of creating recruitment and program materials that were inclusive to both English- and Spanish-speaking people with diabetes living in rural areas was identified. As a result, separate messages and materials were created that were contextually adapted rather than just translated from English to Spanish and included images of the local area and people living in eastern Colorado. The D1D research team learned more about the increased rates of depression and other mood disorders in rural areas, and the C.A.C. learned about the evidence between diabetes and depression or diabetes distress. Changes in content and delivery, as well as supplemental materials, were developed as a result of this shared learning to decrease stigma and increase awareness. Another strategy employed to support the translation of evidence-based guidelines was having a certified dietician review the recipes submitted by the community members. Recipes were to use locally available affordable ingredients, acknowledge—and actually tap into—the local rural and Hispanic and Latino/a cultures, and generally be healthy. However, rather than changing original recipes, the dietician provided suggestions for alternative ingredients and portion sizes, where applicable, which were added to the original recipes. The relationship between people with diabetes and their primary care practice in rural areas was discussed. As a result, a session was added to foster communication with the health care team, as well as development of materials to convey information back to the primary care clinician after R-D1D attendance.

### Limitations

Although other communities, primary care practices, public health educators, and others in rural communities may find these specific results useful, one limitation of these results is that they may not offer broad generalizability. However, the types of adaptations to consider when delivering an intervention in a new community should be more broadly applicable.

### Comparisons With Prior Work

Our results are responsive to calls for more thorough descriptions of the processes and results of community-engaged research activities throughout the research process. Further, this report builds on previous research aiming to more thoroughly describe adaptions to interventions as they are implemented in broad, real-world settings [25-27]. Our findings offer transparent changes to an intervention and can be applied to future R-D1D implementation and dissemination efforts. Telehealth is growing in popularity partially due to its ability to reach populations across broad geographic regions. Although reach is essential, health program researchers, developers, and educators should consider factors that impact program implementation and effectiveness. Patients with diabetes living in rural communities do not have easy access to large endocrinology centers and diabetes care and education specialists and rely heavily on primary care to administer their care. Self-management resources such as the R-D1D program can support patient-provider relationships and effective diabetes management. However, diabetes education in rural eastern Colorado needs to take into account local contextual factors related to rural and Hispanic and Latino/a culture and assets. The R-D1D program development is one example of maximizing medical professional expertise and real-world expertise from people in rural communities and balancing program dissemination goals with “reinventing the wheel” for local relevance [12]. These results matter to patients, families, primary care practices, public health educators, and others in rural communities because good diabetes care should not be dependent on where you live.

### Conclusions

The use of BCT resulted in unique and contextually adapted recruitment and program materials and strategies and changes in program structure and delivery for use in the R-D1D DSMES intervention delivered to English- and Spanish-speaking people with diabetes in rural eastern Colorado, while retaining fidelity to the concepts of the original program. The study team is implementing the R-D1D program. A report on its feasibility and clinical outcomes will be reported separately.

## Appendix

Multimedia Appendix 1 Recruitment and program materials.

## Abbreviations

BCT: Boot Camp Translation
C.A.C.: Community Advisory Council
CDC: Centers for Disease Control and Prevention
D1D: Diabetes One Day
DSMES: diabetes self-management education and support
HbA_1c_: glycated hemoglobin
HPRN: High Plains Research Network
R-D1D: rural Diabetes One Day
T1D: type 1 diabetes
T2D: type 2 diabetes
