# Supplementary material for: Informing a Randomized Control Trial in Rural Populations: Adaptation of a Diabetes Self-Management Education and Support Intervention
Source: JMIR Diabetes. 2022 Jun 10;7(2):e35664. doi: 10.2196/35664 (PMC9233250; doi:10.2196/35664)
Supplement: Multimedia Appendix 1 [file diabetes_v7i2e35664_app1.docx]

| Item | Description | Image |
| --- | --- | --- |
| Participant Study Recruitment Insert | Inserts were printed with culturally-tailored messages about diabetes to promote the Diabetes One-Day program. The message on the English inserts was “You’re Worth It” and was geared towards local farming community members. The message on the Spanish inserts emphasized the importance of family and taking control of diabetes. Inserts were distributed at local businesses and primary care practices. | 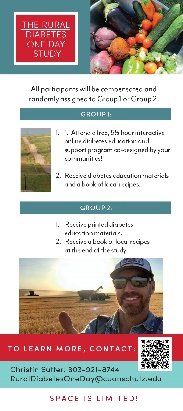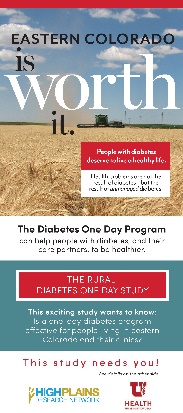  *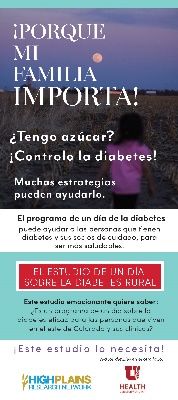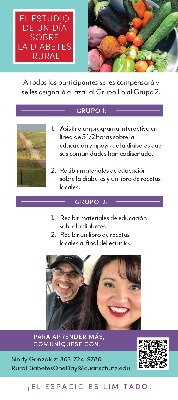English Inserts (front, back)*  *Spanish Inserts (front, back)* |
| Participant Study Recruitment Poster (11” x 17”) | Large print posters with messages about deserving to live a healthy life (English) and living a healthy life for your family (Spanish). Posters were distributed to local primary care practices and various businesses throughout the community to promote the study. | 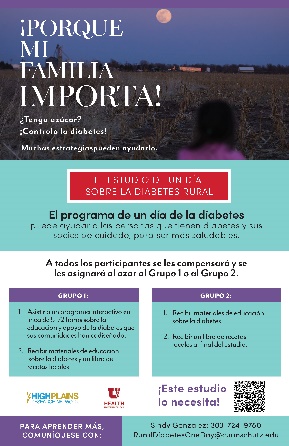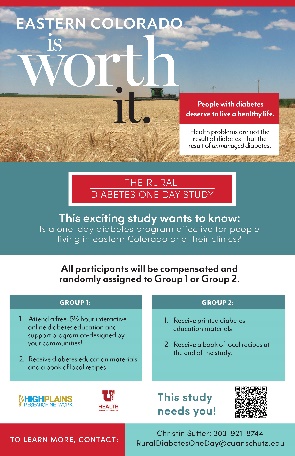*English Poster Spanish Poster* |
| Floor Decals | Decal stickers were place on the floors of clinic waiting room and exam rooms to generate interest in the R-D1D program. | *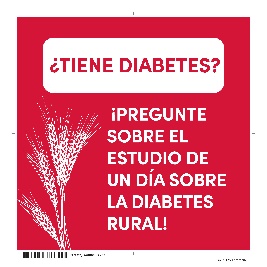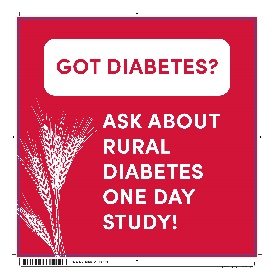*  *English Spanish* |
| Mood tracker | Stickers affixed to the inside cover of small journals to encourage participants to record their mood, feelings, and thoughts about managing diabetes. Available in English and Spanish. | 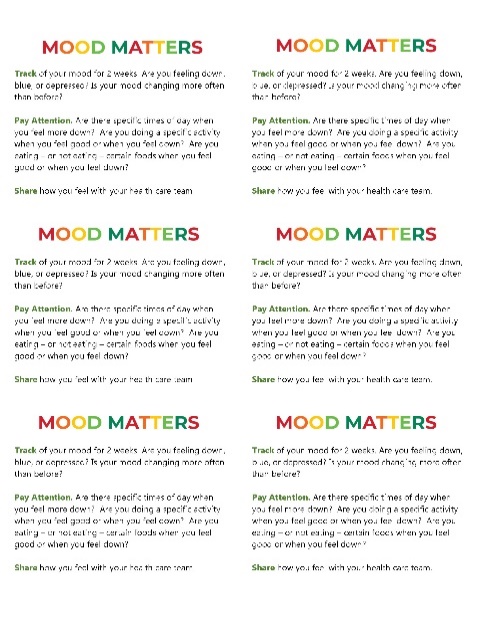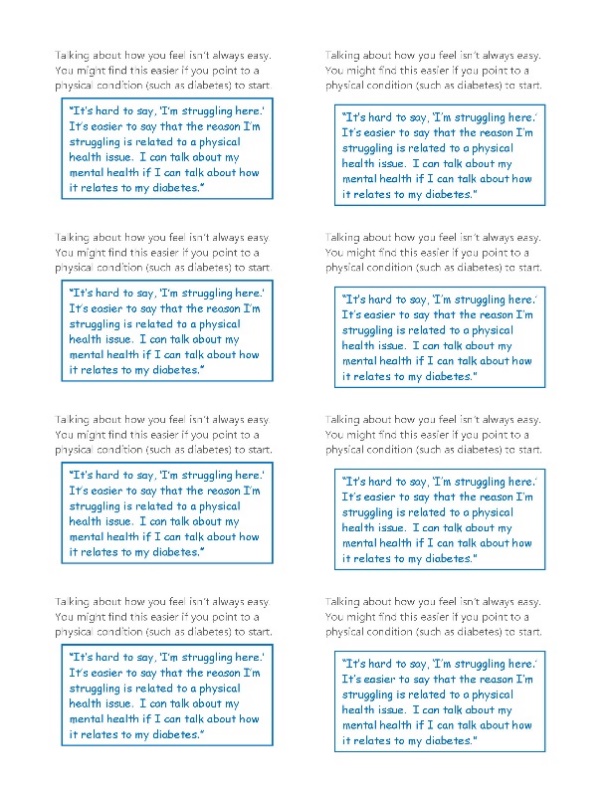 |
| Diabetes, Depression, and Distress Handout | Handout given to participants that attended the R-D1D intervention about the connection between mood, diabetes, and distress. Available in English and Spanish. | 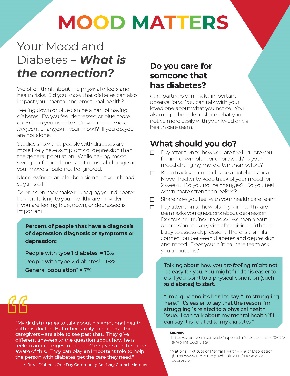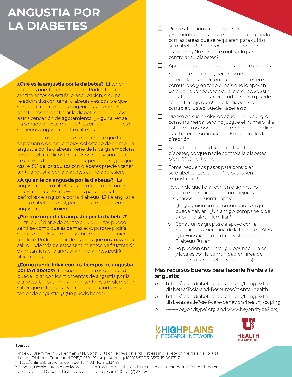 |
| Recipe Book | Recipe book with all recipes submitted by the Community Advisory Council and local community members. Includes tips about portion sizes and healthier choices. Recipe book was distributed to all intervention participants and will be mailed to participants randomized to the control arm after the study. | 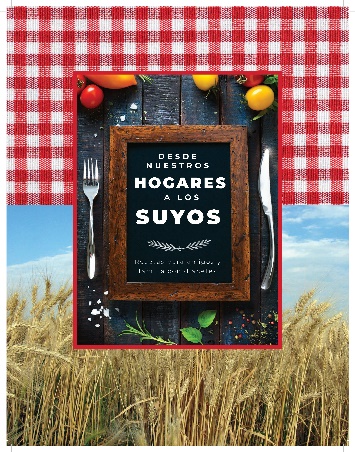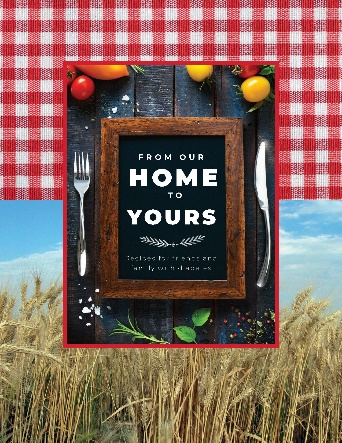  *English Spanish* |
| Cinch bags | Cinch bags with R-D1D logo. Bags held all of the program materials for participants attending the intervention. Available in English and Spanish. | 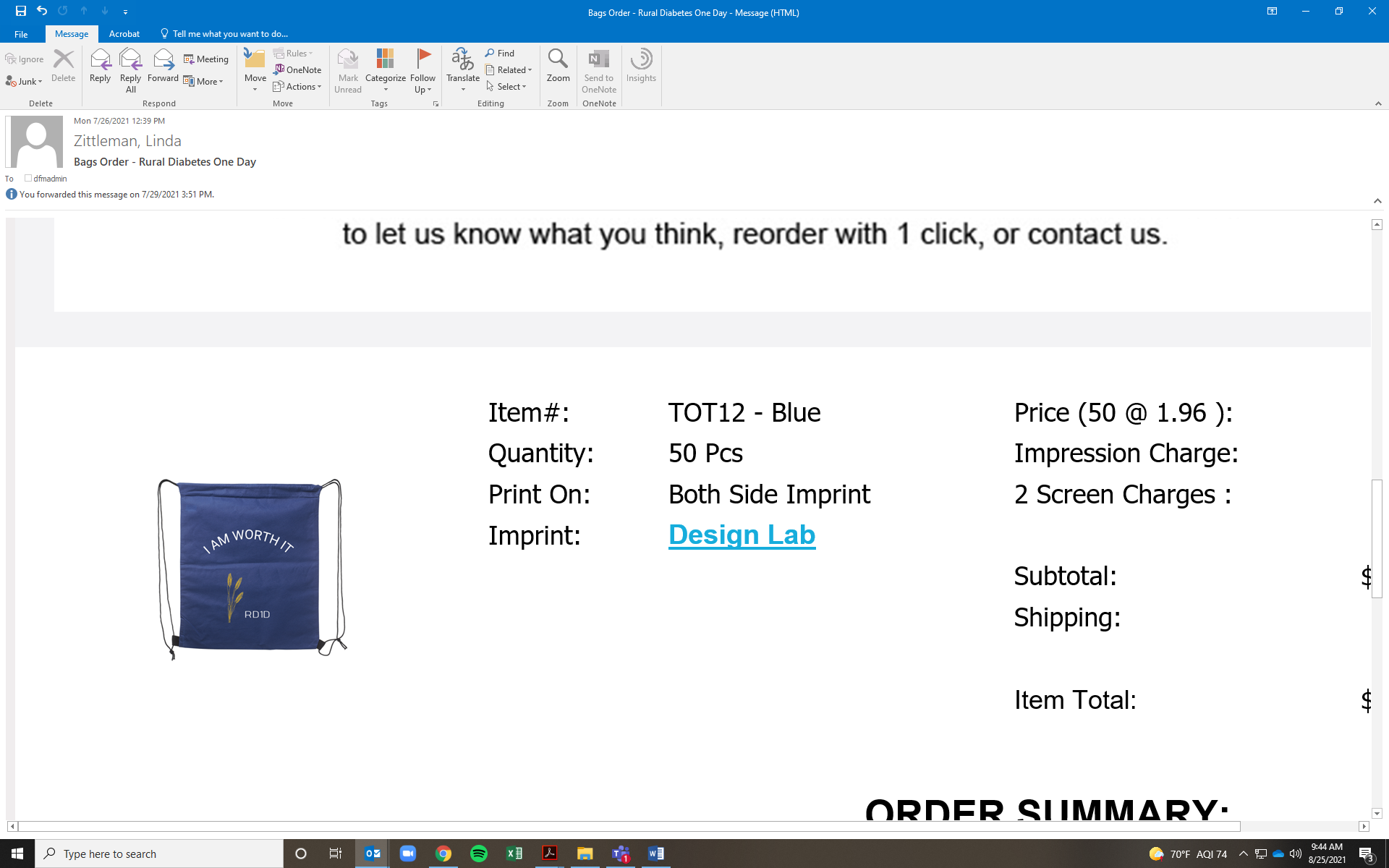 |
| Healthy Plates Placemats | Set of 4 healthy plates placemats were given to participants in the intervention arm. Placemats featured a healthy plate on one side, and one of four photos submitted by a local community member on the other side. Healthy Plate text available in English and Spanish. | *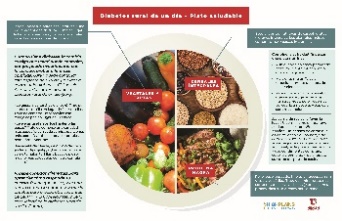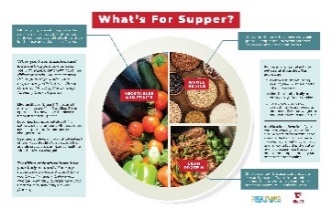*  *English Spanish*  *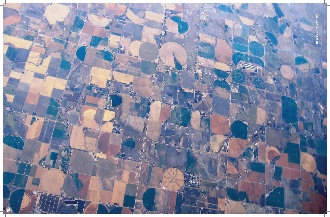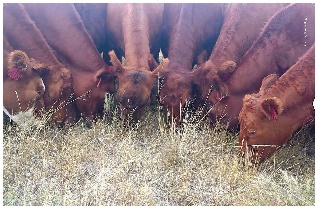*  *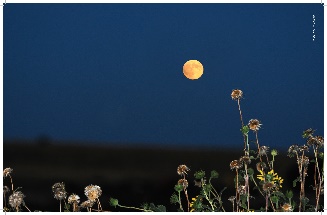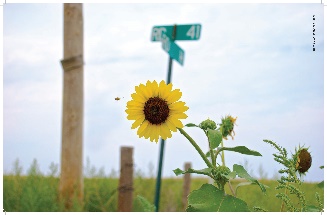Local photography on opposite side of placemat* |
